# Supplementary figures and images for: Targeted distribution of long-lasting insecticidal nets by community health workers to sustain household coverage: A pilot feasibility study in Western Uganda
Source: PLOS Glob Public Health. 2025 Jan 24;5(1):e0003660. doi: 10.1371/journal.pgph.0003660 (PMC11759381; doi:10.1371/journal.pgph.0003660)

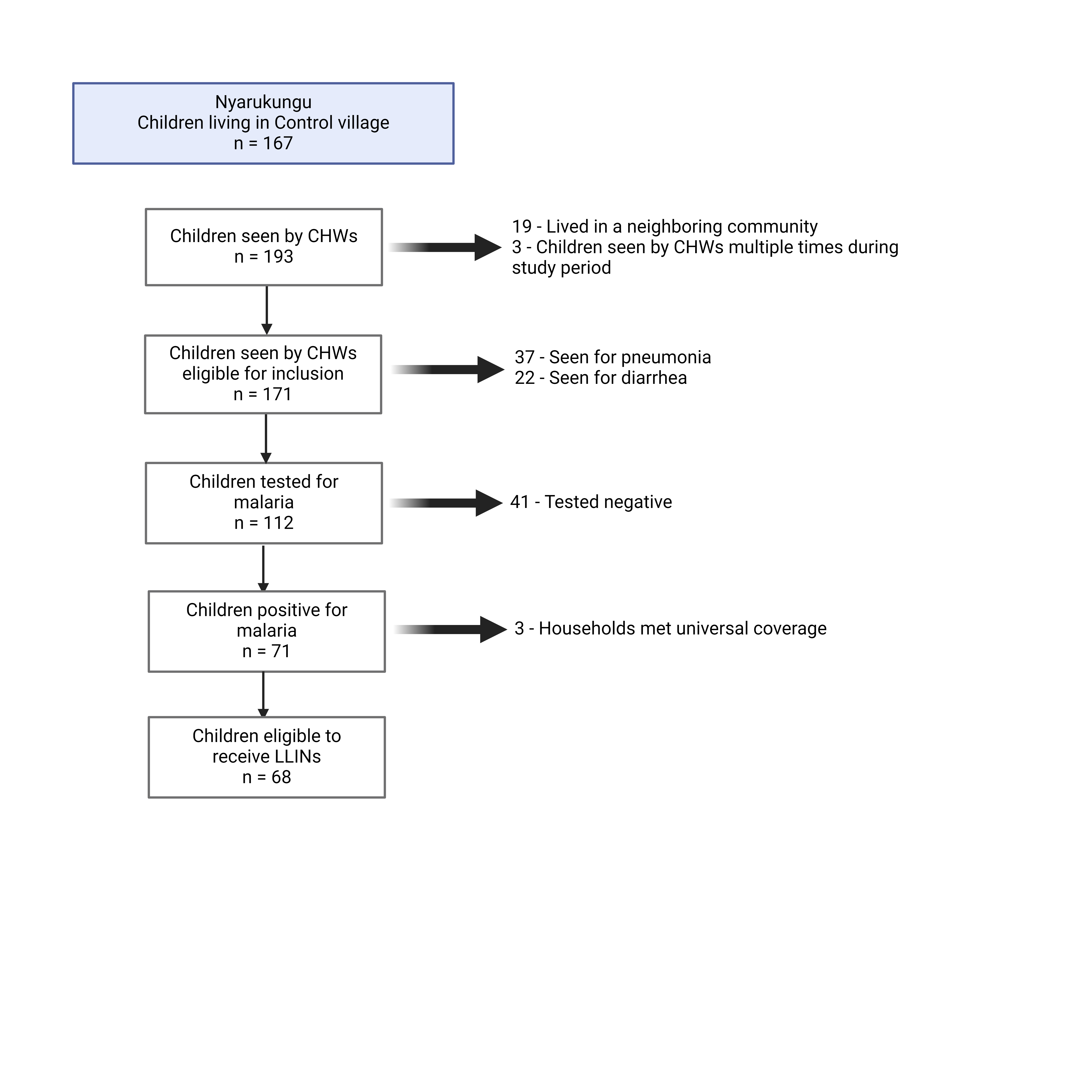

Supplement: S1 Fig — Care cascade indicating the number of children who proceed to each step of the screening process to determine which children are eligible to receive a LLIN through the study in the control village, Nyarukungu. Children meeting each criterion are included in the sample size in each box. Children not meeting the criteria are removed with bolded arrows including the number of children removed and the reason for removal before moving onto the next criterion. The sample size (n) provided in the blue box represents the number of children enumerated from the control village. The starting number of children (n) in the first white box represents the number of visits to a CHW. The number of children enumerated by the study may be an undercount of children living in the communities and was lower than the number of children seen by the CHWs. (TIF) [file pgph.0003660.s001.tif]

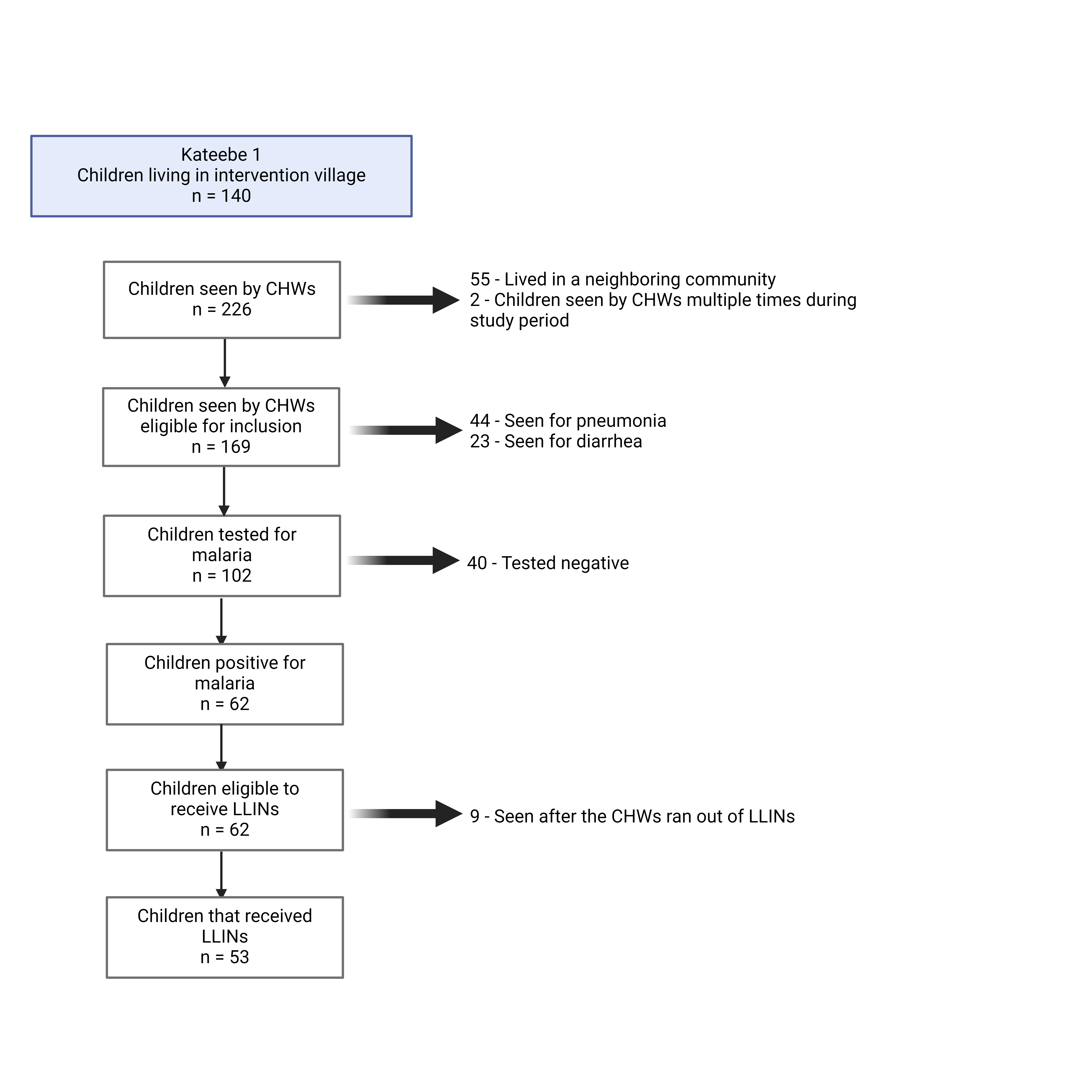

Supplement: S2 Fig — Care cascade indicating the number of children who proceed to each step of the screening process to determine which children would receive a LLIN through the study in the intervention village, Kateebe 1. Children meeting each criterion are included in the sample size in each box. Children not meeting the criteria are removed with bolded arrows including the number of children removed and the reason for removal before moving onto the next criterion. The sample size (n) provided in the blue box represents the number of children enumerated from the intervention village. The starting number of children (n) in the first white box represents the number of visits to a CHW. The number of children enumerated by the study may be an undercount of children living in the communities and was lower than the number of children seen by the CHWs. (TIF) [file pgph.0003660.s002.tif]
