# Supplementary material for: Targeted distribution of long-lasting insecticidal nets by community health workers to sustain household coverage: A pilot feasibility study in Western Uganda
Source: PLOS Glob Public Health. 2025 Jan 24;5(1):e0003660. doi: 10.1371/journal.pgph.0003660 (PMC11759381; doi:10.1371/journal.pgph.0003660)
Supplement: S1 Table — This table provides counts and percentages for missing values out of all eligible participants per cell for the description of the study population as presented in Table 1. Percentages for missing data on marriage status and education level are calculated from all adults in the study, not just the head of the household and second adult as in Table 1. (DOCX) [file pgph.0003660.s003.docx]

|  | **Kateebe 1**  Intervention  (N (%)) | **Nyarukungu** Control  (N (%)) | Total  (N (%)) |
| --- | --- | --- | --- |
|  | 153 Households | 180 Households | 333 Households |
| Age^1^ | | |  |
| Adults (≥18 years) | 363 | 417 | 780 |
| Children | | | |
| Age 13 to 18 years | 114 | 191 | 305 |
| Age 5 to 12 years | 234 | 236 | 470 |
| Age <5 years | 140 | 167 | 307 |
| Household Measures | | | |
| Earthen or sand floor | 3 (2.0) | 2 (1.1) | 5 (1.5) |
| Electricity in the house | 1 (0.7) | 1 (0.6) | 2 (0.6) |
| Access to piped water | 1 (0.7) | 1 (0.6) | 2 (0.6) |
| Own a mobile phone | 1 (0.7) | 2 (1.1) | 3 (0.9) |
| Own livestock | 1 (0.7) | 2 (1.1) | 3 (0.9) |
| Have a bank account | 1 (0.7) | 4 (2.2) | 5 (1.5) |
| Adults | | | |
| Age | 1 (0.3) | 4 (1.0) | 5 (0.6) |
| Male | 10 (2.8) | 9 (2.2) | 19 (2.4) |
| Married | 68 (18.7) | 75 (18.0) | 143 (18.3) |
| Highest level of school | 68 (18.7) | 74 (17.7) | 142 (18.2) |
| Children^2^ | | | |
| Age | 2 (0.5) | 3 (0.7) | 5 (0.6) |
| Male | 25 (6.7) | 33 (8.2) | 58 (7.5) |
| Malnourished (MUAC <14.3) ^3^ | 49 (35.0) | 63 (37.8) | 112 (36.5) |
| Birth location | 25 (6.7) | 32 (7.9) | 57 (7.3) |
| Hospitalized in the last 12 months from malaria (Children < 18) | 2 (0.5) | 6 (1.5) | 8 (1.0) |

*Table S1: Missing Data for Population Demographics.* This table provides counts and percentages for missing values out of all eligible participants per cell for the description of the study population as presented in table 1. Percentages for missing data on marriage status and education level are calculated from all adults in the study, not just the head of the household and second adult as in table 1.

^1^ Age section does not include missing data, there are for reference for missing data rows in subsequent sections of the table.

^2^ Only includes children 12 years old and younger.

^3^ MUAC was only measured for children 2 to 5 years old.
