# Supplementary material for: Targeted distribution of long-lasting insecticidal nets by community health workers to sustain household coverage: A pilot feasibility study in Western Uganda
Source: PLOS Glob Public Health. 2025 Jan 24;5(1):e0003660. doi: 10.1371/journal.pgph.0003660 (PMC11759381; doi:10.1371/journal.pgph.0003660)
Supplement: S2 Table — This table provides counts and percentages for missing values out of all eligible participants per cell for individuals and households before and after the intervention in both the intervention (Kateebe 1) and control villages (Nyarukungu). Some data pertain only to the post intervention timepoint, and, therefore, were not applicable (NA) at the pre intervention timepoint. Total counts were included along with percentages in parenthesis. Percentages for adults, total children, and specific age groups of children are out of all participants. Percentages of children tested for malaria are out of children aged 2 to 10 years old. (DOCX) [file pgph.0003660.s004.docx]

S2 Table: Missing Data for Pre-Post intervention data.

|  | **Kateebe 1**  Intervention Village  (N (%)) | | **Nyarukungu**  Control Village  (N (%)) | |
| --- | --- | --- | --- | --- |
|  | Pre | Post | Pre | Post |
| Sample Sizes^1^ | | | | |
| Households | 153 | 109 | 180 | 149 |
| Total individuals | 837 | 720 | 1007 | 951 |
| Adults | 363 | 303 | 417 | 379 |
| Children (total) | 474 | 417 | 590 | 572 |
| Children aged 2 to 10 | 278 | 234 | 300 | 300 |
| Children aged 2 to 10 tested for malaria (PfPR)^2^ | 233 | 153 | 250 | 260 |
| Children under the age of 5 | 140 | 122 | 167 | 158 |
| Among all households | | | | |
| Level of coverage | 1 (0.7) | 1 (0.9) | 2 (0.6) | 1 (0.7) |
| Households reporting incident malaria infection during study period (any households) | NA | 2 (1.8) | NA | 1 (0.7) |
| PfPR among children 2-10 years of age^2,3^ | 0 (0) | 0 (0) | 0 (0) | 0 (0) |
| Among household that have LLINs: | | | | |
| Proportion of household members who slept the prior night under a LLIN | 0 (0) | 2 (1.8) | 0 (0) | 2 (1.3) |
| Reasons why household members did not sleep under an LLIN^4^ | 0 (0) | 0 (0) | 0 (0) | 0 (0) |
| Proportion of household LLINs with major damage (median (IQR)) ^5^ | 0 (0) | 0 (0) | 0 (0) | 0 (0) |
| Proportion of LLINs were used last night | 2 (5.4) | 2 (1.8) | 14 (25.5) | 2 (1.3) |
| Under-fives who slept the prior night under a LLIN | 13 (9.3) | 8 (6.6) | 16 (9.0) | 15 (9.5) |
| Households receiving new LLINs from any source | NA | 0 (0) | NA | 0 (0) |
| Number of LLINs received from CHW | NA | 0 (0) | NA | 0 (0) |
| Incident malaria infection during study period (since receipt of new LLINs) | NA | 0 (0) | NA | 0 (0) |

This table provides counts and percentages for missing values out of all eligible participants per cell for individuals and households before and after the intervention in both the intervention (Kateebe 1) and control villages (Nyarukungu). Some data pertain only to the post intervention timepoint, and, therefore, were not applicable (NA) at the pre intervention timepoint. Total counts were included along with percentages in parenthesis. Percentages for adults, total children, and specific age groups of children are out of all participants. Percentages of children tested for malaria are out of children aged 2 to 10 years old.

^1^ Sample sizes do not include missing data, there are for reference for missing data rows in subsequent sections of the table

^2^ PfPR - Plasmodium falciparum parasite rate

^3^ Only includes missing data among those children actually tested. Children not being tested even though they were eligible, it is due to their absence from the home when researchers visited the homes. This includes 45 children in Kateebe 1 and 50 in Nyarukungu from the pre survey and includes 81 children in Kateebe 1 and 40 in Nyarukungu from the post survey.

^4^ More than one answer could be selected by households. Only includes households that had LLINs and household members that did not sleep under an LLIN

^5^ Major damage is defined as no tears or holes >2cm (i.e., larger than thumb)
