# Supplementary material for: Targeted distribution of long-lasting insecticidal nets by community health workers to sustain household coverage: A pilot feasibility study in Western Uganda
Source: PLOS Glob Public Health. 2025 Jan 24;5(1):e0003660. doi: 10.1371/journal.pgph.0003660 (PMC11759381; doi:10.1371/journal.pgph.0003660)
Supplement: S1 Appendix — (PDF) [file pgph.0003660.s005.pdf]

Enrollment Form

REDCap #	______________________________ (Automatic Pull)
Village	◯ Kateebe 1<br>◯ Nyarukungu
Study Number<br><br>Enter the designator for the village name (Kateebe K, Nyarunkungu = N) followed by the RA assigned number (e.g. 100, 200, etc), followed by the number in that village. This number should correspond to the Study ID card given to the head-of-household.<br><br>Examples:<br>1. The third house visited by RA #2 in Kateebe 1 = K203<br>2. The twenty seventh house visited by RA #4 in Nyarunkungu = N427	______________________________
Date of Consent	______________________________
Are there any children (age 2 - 10 years) in the house?	◯ Yes<br>◯ No
RA Documenting Consent	◯ Mugisa Saul<br>◯ Kibaba Georget<br>◯ Bwambale Jonathan<br>◯ Bwambale Aprunale<br>◯ Biira Doreen<br>◯ Asikibawe Norbert<br>◯ Lyhinda Francis<br>◯ Baluku Jockim
Comments	______________________________________

Household Information

REDCap #

______________________ (Automatic Pull)

Household Location

Attempt to pull GPS location from REDCap. If not able to locate, then use install ed app and manually enter data below.

Latitude

____

Longitude

____

Did latitude and longitude successfully enter?
- ◯ Yes
- ◯ No

Enter manual latitude

______________________ (Ex. 0.XXXXX)

Confirm manual latitude

______________________ (Ex. 0.XXXXX)

VALUES DO NOT MATCH

Enter manual longitude

______________________ (30.YYYYY)

Confirm manual longitude

______________________ (Ex. 30.YYYYY)

VALUES DO NOT MATCH

Who is your current or most recent VHT? (Kateebe 1)
- ◯ Mughanda Herimon
- ◯ Biira Jackline
- ◯ Baluku Edson Baghuma
- ◯ Biira Miria
- ◯ Kisembo Charles

(select one)

Who is your current (or most recent) VHT? (Nyarukungu)
- ◯ Kabugho Naume
- ◯ Musoki Scovia
- ◯ Kighoma Elisha
- ◯ Buhaka Grace
- ◯ Kabugho Grace

(select one)

Household Characteristics

Ask participant about these features of the household and markers of household wealth

What is the main source of drinking water for members of your household?	◯ Piped water<br>◯ Tube well or borehole<br>◯ Dug well<br>◯ Water from spring<br>◯ Rainwater<br>◯ Tanker truck<br>◯ Bicycle with jerrycans<br>◯ Surface water (river, lake, stream, irrigation channel)<br>◯ Bottled water<br>◯ Other
Where is the water piped:	◯ Piped into house<br>◯ Piped to yard/plot<br>◯ Piped to neighbor<br>◯ Piped to public tap
If a dug well, please specify the type:	◯ Unprotected well<br>◯ Protected well
If other source of water, please specify	________________________________
Where is that water source located?	◯ In own dwelling<br>◯ In own yard or plot<br>◯ Elsewhere
How many minutes does it take to get there, get water, and come back?	__________________________<br>(Minutes)
Do you do anything to the water to make it safer to drink?	◯ Yes<br>◯ No
What do you usually do to make the water safer to drink?	☐ Boil<br>☐ Add bleach or chlorine<br>☐ Strain through a cloth<br>☐ Use water filter<br>☐ Solar disinfection<br>☐ Let is stand and settle<br>☐ Other
If other method of treating water, please specify:	________________________________
What kind of toilet facility do members of your household usually use?<br><br>IF NOT POSSIBLE TO DETERMINE, ASK PERMISSION TO OBSERVE THE FACILITY.	◯ Flush or pour toilet<br>◯ Pit Latrine<br>◯ Composting toilet<br>◯ Bucket toilet<br>◯ Hanging toilet<br>◯ No facility/bush<br>◯ Other

If other type of toilet, please specify:	____________________
Do you share this toilet facility with other households?	◯ Yes<br>◯ No
Including your own household, how many other households use this toilet facility?	____________________
Where is the toilet located?	◯ In own dwelling<br>◯ In own yard or plot<br>◯ Elsewhere
How many minutes does it take to get to the latrine and come back?	____________________<br>(Minutes)
What type of fuel does your household mainly use for cooking?	◯ Electricity<br>◯ LPG/Cylinder Gas<br>◯ Biogas<br>◯ Kerosene<br>◯ Charcoal<br>◯ Wood<br>◯ Straw/Grass<br>◯ Agricultural crop<br>◯ Animal dung<br>◯ No food cooked in household<br>◯ Other
If other type of fuel, please specify:	____________________
Where is the cooking of food usually done?	◯ In the house<br>◯ In a separate building<br>◯ Outdoors<br>◯ Other
If cooking is done somewhere else, please specify:	____________________
How many rooms are in this household?	____________________
Do you have a separate room which is used as a kitchen?	◯ Yes<br>◯ No
How many rooms in this household are used for sleeping?	____________________
Does this household own any livestock, herds, other farm animals, or poultry?	◯ Yes<br>◯ No

If yes, what type of livestock:	☐ Cattle<br>☐ Goats<br>☐ Sheep<br>☐ Chickens<br>☐ Pigs<br>(Check all that apply)
How many cattle?	____________________
How many goats?	____________________
How many sheep?	____________________
How many chickens?	____________________
How many pigs?	____________________
Are there any animals that sleep in the house where people sleep?	◯ Yes<br>◯ No
Does your household have:	☐ Electricity<br>☐ Radio<br>☐ Television<br>☐ Computer<br>☐ Refrigerator<br>☐ Casette/CD/DVD player<br>☐ Table<br>☐ Chair<br>☐ Bed<br>☐ Cupboard<br>☐ Clock<br>(Check all that apply)
Does any member of this household own:	☐ Watch<br>☐ Mobile phone<br>☐ Bicycle<br>☐ Motorcycle<br>☐ Car or truck<br>(Check all that apply)
Does any member of this household have a bank account, mobile money account, or account with an agent?	◯ Yes<br>◯ No

Direct Observations of General House Construction

With the participants permission, inspect the design of the house. When construction materials are mixed, then select the answer that best characterizes the majority of the home.

Observe the main material of the floor of the dwelling. Record observation.

- ◯ Natural floor - earth/sand
- ◯ Basic floor - wood planks
- ◯ Basic floor - palm/bamboo
- ◯ Finished floor - polished wood
- ◯ Finished floor - concrete
- ◯ Finished floor - tile
- ◯ Finished floor - stones
- ◯ Finished floor - brick
- ◯ Other

If other type of floor, please specify:

____

Observe the main material of the roof of the dwelling. Record observation.

- ◯ No roof
- ◯ Natural roof - thatch / palm leaf
- ◯ Natural roof - mud
- ◯ Basic roof - mat
- ◯ Basic roof - tin
- ◯ Basic roof - wood planks
- ◯ Basic roof - cardboard
- ◯ Basic roof - plastic tarpaulin
- ◯ Finished roof - iron sheets
- ◯ Finished roof - wood
- ◯ Finished roof - asbestos
- ◯ Finished roof - tiles
- ◯ Finished roof - concrete
- ◯ Finished roof - roofing shingles
- ◯ Other

If other type of roof, please specify:

____

Observe the main material of the exterior walls of the dwelling. Record observation.

- ◯ No walls
- ◯ Natural walls - thatched/straw
- ◯ Natural walls - dirt
- ◯ Basic walls - poles with mud
- ◯ Basic walls - stone with mud
- ◯ Basic walls - unburnt bricks with mud
- ◯ Basic walls - plywood
- ◯ Basic walls - cardboard
- ◯ Basic walls - reused wood
- ◯ Basic walls - unburnt bricks with plaster
- ◯ Basic walls - burnt bricks with mud
- ◯ Finished walls - cement
- ◯ Finished walls - stone with lime/cement
- ◯ Finished walls - burnt bricks with cement
- ◯ Finished walls - cement blocks
- ◯ Finished walls - unburnt bricks with cement
- ◯ Finished walls - wood planks/shingles
- ◯ Other

If other type of walls, please specify:

____

Direct Observation of Sleeping Room

Explain to the participant that malaria is often transmitted when mosquitoes bite people at night while they are sleeping and therefore it is important to document sleeping conditions.

With the participants permission, inspect features of the main sleeping room (i.e. bedroom). If multiple sleeping rooms are present, then observe the room where the eligible child sleeps.

Is there a door in the main room that is used for sleeping that leads outside?	◯ Yes<br>◯ No
Do the household members sleep in a bed?	◯ Yes<br>◯ No
Is there a window in the main room that is used for sleeping	◯ Yes<br>◯ No
If there is a window, does it close completely or have screening?	◯ Yes<br>◯ No
Are there eaves (space between the roof and wall) in the main room that is used for sleeping?	◯ Yes<br>◯ No
If there are eaves, do they have screening?	◯ Yes<br>◯ No
Are there bricks that allow air into the main room that is used for sleeping?	◯ Yes<br>◯ No
If there are air bricks, are they covered with screen?	◯ Yes<br>◯ No
If there are air bricks in the main room that is used for sleeping, how many?	____________________________
Comments	____________________________________

Household Members & Testing

REDCap #

____
(Automatic Pull)

Household Members

Include any individuals who spend most nights of the week in the house.

How many individuals live in this household? ______________________________

How many adults (age ≥ 18 years) live in this household? ______________________________

How many older children (age 13 to 18 years) live in this household? ______________________________

How many children (age 5 to 12 years) live in this household? ______________________________

How many children (age less than 5 years) live in this household? ______________________________

Adult Household Members

Ask information about adult members of the household. The male and female head-of-household (i.e. husband/wife or father/mother) should be Adult #1 and Adult #2. Adult children (age ≥18 years) of the head-of-household should also be included here if they still live in the house.

Questions are available for up to six adult household members. If more than six adults live in the household, then record information about six, then document additional information in Comments section at bottom of form.

Sex of Adult #1

- ◯ Female
- ◯ Male

Age of Adult #1

____
(Years)

What category best describes Adult #1

- ◯ Mother
- ◯ Father
- ◯ Adult child
- ◯ Grandmother
- ◯ Grandfather
- ◯ Other

If other, please specify:

____

What is the highest level of school Adult #1 completed?

- ◯ No school
- ◯ Primary School
- ◯ Secondary School
- ◯ University

What is the marital status of Adult #1?

- ◯ Unmarried
- ◯ Married
- ◯ Divorced
- ◯ Widowed

Sex of Adult #2

- ◯ Female
- ◯ Male

Age of Adult #2

____
(Years)

What category best describes Adult #2

- ◯ Mother
- ◯ Father
- ◯ Adult child
- ◯ Grandmother
- ◯ Grandfather
- ◯ Other

If other, please specify:

____

What is the highest level of school Adult #2 completed?

- ◯ No school
- ◯ Primary School
- ◯ Secondary School
- ◯ University

What is the marital status of Adult #2?

- ◯ Unmarried
- ◯ Married
- ◯ Divorced
- ◯ Widowed

Sex of Adult #3

- ◯ Female
- ◯ Male

Age of Adult #3

____
(Years)

What category best describes Adult #3

- ◯ Mother
- ◯ Father
- ◯ Adult child
- ◯ Grandmother
- ◯ Grandfather
- ◯ Other

If other, please specify:	______________________
Sex of Adult #4	◯ Female<br>◯ Male
Age of Adult #4	______________________<br>(Years)
What category best describes Adult #4	◯ Mother<br>◯ Father<br>◯ Adult child<br>◯ Grandmother<br>◯ Grandfather<br>◯ Other
If other, please specify:	______________________
Sex of Adult #5	◯ Female<br>◯ Male
Age of Adult #5	______________________<br>(Years)
What category best describes Adult #5	◯ Mother<br>◯ Father<br>◯ Adult child<br>◯ Grandmother<br>◯ Grandfather<br>◯ Other
If other, please specify:	______________________
Sex of Adult #6	◯ Female<br>◯ Male
Age of Adult #6	______________________<br>(Years)
What category best describes Adult #6	◯ Mother<br>◯ Father<br>◯ Adult child<br>◯ Grandmother<br>◯ Grandfather<br>◯ Other
If other, please specify:	______________________

Additional Comments

____

Child Information

Include information on all children 17 years and younger who live in the household. Enter data from youngest child to oldest child. For example, Child #1 should be the oldest child.

If more than 6 children live in the household, document additional information in the Comments section below.

Sex of Child #1 (Oldest)	◯ Female<br>◯ Male
Age of Child #1<br><br>IF CHILD IS LESS THAN 1 YEAR, ENTER 0 WHICH WILL OPEN NEW QUESTION ABOUT MONTHS	_____________________<br>(Years)
If Child #1 is less than 1 year of age, how many months old is the child	_____________________<br>(Months)
Where was Child #1 born?	◯ Home<br>◯ Government Health Facility<br>◯ Government Hospital<br>◯ Private Clinic or Hospital<br>◯ Other
If other, please list here:	__________________________
Did Child #1 sleep under a bed net last night?	◯ Yes<br>◯ No
Has Child #1 stayed overnight in a hospital or clinic for malaria in the past year?	◯ Yes<br>◯ No
Child #1 temperature:	_____________________<br>(Degrees Celsius)
Child #1 Mid-Upper Arm Circumference (MUAC)<br><br>ONLY FOR CHILDREN 2 to 5 YEARS OF AGE	_____________________<br>(centimeters)
Child #1 Malaria RDT Result:	◯ Negative<br>◯ Positive<br>◯ Invalid / Unable to Perform
Sex of Child #2	◯ Female<br>◯ Male

Age of Child #2<br><br>IF CHILD IS LESS THAN 1 YEAR, ENTER 0 WHICH WILL OPEN NEW QUESTION ABOUT MONTHS	______________________<br>(Years)
If Child #2 is less than 1 year of age, how many months old is the child	______________________<br>(Months)
Where was Child #2 born?	◯ Home<br>◯ Government Health Facility<br>◯ Government Hospital<br>◯ Private Clinic or Hospital<br>◯ Other
If other, please list here:	____________________________
Did Child #2 sleep under a bed net last night?	◯ Yes<br>◯ No
Has Child #2 stayed overnight in a hospital or clinic for malaria in the past year?	◯ Yes<br>◯ No
Child #2 temperature:	______________________<br>(Degrees Celsius)
Child #2 Mid-Upper Arm Circumference (MUAC)<br><br>ONLY FOR CHILDREN 2 to 5 YEARS OF AGE	______________________<br>(centimeters)
Child #2 Malaria RDT Result:	◯ Negative<br>◯ Positive<br>◯ Invalid / Unable to Perform
Sex of Child #3	◯ Female<br>◯ Male
Age of Child #3<br><br>IF CHILD IS LESS THAN 1 YEAR, ENTER 0 WHICH WILL OPEN NEW QUESTION ABOUT MONTHS	______________________<br>(Years)
If Child #3 is less than 1 year of age, how many months old is the child	______________________<br>(Months)
Where was Child #3 born?	◯ Home<br>◯ Government Health Facility<br>◯ Government Hospital<br>◯ Private Clinic or Hospital<br>◯ Other

If other, please list here:	______________________
Did Child #3 sleep under a bed net last night?	◯ Yes<br>◯ No
Has Child #3 stayed overnight in a hospital or clinic for malaria in the past year?	◯ Yes<br>◯ No
Child #3 temperature:	______________________<br>(Degrees Celsius)
Child #3 Mid-Upper Arm Circumference (MUAC)<br><br>ONLY FOR CHILDREN 2 to 5 YEARS OF AGE	______________________<br>(centimeters)
Child #3 Malaria RDT Result:	◯ Negative<br>◯ Positive<br>◯ Invalid / Unable to Perform
Sex of Child #4	◯ Female<br>◯ Male
Age of Child #4<br><br>IF CHILD IS LESS THAN 1 YEAR, ENTER 0 WHICH WILL OPEN NEW QUESTION ABOUT MONTHS	______________________<br>(Years)
If Child #4 is less than 1 year of age, how many months old is the child	______________________<br>(Months)
Where was Child #4 born?	◯ Home<br>◯ Government Health Facility<br>◯ Government Hospital<br>◯ Private Clinic or Hospital<br>◯ Other
If other, please list here:	______________________
Did Child #4 sleep under a bed net last night?	◯ Yes<br>◯ No
Has Child #4 stayed overnight in a hospital or clinic for malaria in the past year?	◯ Yes<br>◯ No
Child #4 temperature:	______________________<br>(Degrees Celsius)
Child #4 Mid-Upper Arm Circumference (MUAC)<br><br>ONLY FOR CHILDREN 2 to 5 YEARS OF AGE	______________________<br>(centimeters)

Child #4 Malaria RDT Result:	◯ Negative<br>◯ Positive<br>◯ Invalid / Unable to Perform
Sex of Child #5	◯ Female<br>◯ Male
Age of Child #5<br><br>IF CHILD IS LESS THAN 1 YEAR, ENTER 0 WHICH WILL OPEN NEW QUESTION ABOUT MONTHS	__________________<br>(Years)
If Child #5 is less than 1 year of age, how many months old is the child	__________________<br>(Months)
Where was Child #5 born?	◯ Home<br>◯ Government Health Facility<br>◯ Government Hospital<br>◯ Private Clinic or Hospital<br>◯ Other
If other, please list here:	______________________________
Did Child #5 sleep under a bed net last night?	◯ Yes<br>◯ No
Has Child #5 stayed overnight in a hospital or clinic for malaria in the past year?	◯ Yes<br>◯ No
Child #5 temperature:	__________________<br>(Degrees Celsius)
Child #5 Mid-Upper Arm Circumference (MUAC)<br><br>ONLY FOR CHILDREN 2 to 5 YEARS OF AGE	__________________<br>(centimeters)
Child #5 Malaria RDT Result:	◯ Negative<br>◯ Positive<br>◯ Invalid / Unable to Perform
Sex of Child #6	◯ Female<br>◯ Male
Age of Child #6<br><br>IF CHILD IS LESS THAN 1 YEAR, ENTER 0 WHICH WILL OPEN NEW QUESTION ABOUT MONTHS	__________________<br>(Years)
If Child #6 is less than 1 year of age, how many months old is the child	__________________<br>(Months)

Where was Child #6 born?	◯ Home<br>◯ Government Health Facility<br>◯ Government Hospital<br>◯ Private Clinic or Hospital<br>◯ Other
If other, please list here:	________________________________
Did Child #6 sleep under a bed net last night?	◯ Yes<br>◯ No
Has Child #6 stayed overnight in a hospital or clinic for malaria in the past year?	◯ Yes<br>◯ No
Child #6 temperature:	________________________<br>(Degrees Celsius)
Child #6 Mid-Upper Arm Circumference (MUAC)<br><br>ONLY FOR CHILDREN 2 to 5 YEARS OF AGE	________________________<br>(centimeters)
Child #6 Malaria RDT Result:	◯ Negative<br>◯ Positive<br>◯ Invalid / Unable to Perform
Additional Comments	________________________________

Malaria Prevention & Treatment_Baseline

REDCap # ______________________ (Automatic Pull)

Insecticide Treated Nets

Does your household have any mosquito nets?
- ◯ Yes
- ◯ No

How many mosquito nets does your household have? ______________________

How many mosquito nets were used last night? ______________________

How many household members slept under a mosquito net last night? ______________________

Did any household members NOT sleep under a mosquito net last night?
- ◯ Yes
- ◯ No

Please list the age and sex of any household members who did NOT sleep under a mosquito net last night ______________________

Why did these members not sleep under a net?
- ☐ Not enough nets available
- ☐ Net has too many holes or is damaged
- ☐ Too hot, uncomfortable, or don't like smell
- ☐ No place to hang or place it
- ☐ Not many mosquitoes / Low risk for malaria
- ☐ Other

(Select all that apply)

If other reasons stated for not using a mosquito net, please list here: ______________________

For Net #1, what was the source of this net?
- ◯ Received from government distribution
- ◯ Received at a health facility
- ◯ Purchased from store
- ◯ Other

If Net #1 was obtained from another source, please specify: ______________________

For Net #1, how old is this net:

ASK PARTICIPANT AND ATTEMPT TO CONFIRM ON TAG
- ◯ Less than one year old
- ◯ One to two years old
- ◯ More than two years old

Does Net #1 have any holes?
- ◯ Yes
- ◯ No

Net #1 Condition

	0	1	2	3	4	≥ 5
Holes 0.5 to 2 cm (thumb size)	◯	◯	◯	◯	◯	◯
Holes 2 to 10 cm (bigger than thumb, smaller than fist)	◯	◯	◯	◯	◯	◯
Holes 10 to 25 cm (bigger than first, smaller than head)	◯	◯	◯	◯	◯	◯
Holes >25 cm (head size)	◯	◯	◯	◯	◯	◯

For Net #2, what was the source of this net?

- ◯ Received from government distribution
- ◯ Received at a health facility
- ◯ Purchased from store
- ◯ Other

If Net #2 was obtained from another source, please specify:

____

For Net #2, how old is this net:

ASK PARTICIPANT AND ATTEMPT TO CONFIRM ON TAG

- ◯ Less than one year old
- ◯ One to two years old
- ◯ More than two years old

Does Net #2 have any holes?

- ◯ Yes
- ◯ No

Net #2 Condition

	0	1	2	3	4	≥ 5
Holes 0.5 to 2 cm (thumb size)	◯	◯	◯	◯	◯	◯
Holes 2 to 10 cm (bigger than thumb, smaller than fist)	◯	◯	◯	◯	◯	◯
Holes 10 to 25 cm (bigger than first, smaller than head)	◯	◯	◯	◯	◯	◯
Holes >25 cm (head size)	◯	◯	◯	◯	◯	◯

For Net #3, what was the source of this net?

- ◯ Received from government distribution
- ◯ Received at a health facility
- ◯ Purchased from store
- ◯ Other

If Net #3 was obtained from another source, please specify:

____

For Net #3, how old is this net:

ASK PARTICIPANT AND ATTEMPT TO CONFIRM ON TAG

- ◯ Less than one year old
- ◯ One to two years old
- ◯ More than two years old

Does Net #3 have any holes?

- ◯ Yes
- ◯ No

Net #3 Condition

	0	1	2	3	4	≥ 5
Holes 0.5 to 2 cm (thumb size)	◯	◯	◯	◯	◯	◯
Holes 2 to 10 cm (bigger than thumb, smaller than fist)	◯	◯	◯	◯	◯	◯
Holes 10 to 25 cm (bigger than first, smaller than head)	◯	◯	◯	◯	◯	◯
Holes >25 cm (head size)	◯	◯	◯	◯	◯	◯

For Net #4, what was the source of this net?

- ◯ Received from government distribution
- ◯ Received at a health facility
- ◯ Purchased from store
- ◯ Other

If Net #4 was obtained from another source, please specify:

____

For Net #4, how old is this net:

ASK PARTICIPANT AND ATTEMPT TO CONFIRM ON TAG

- ◯ Less than one year old
- ◯ One to two years old
- ◯ More than two years old

Does Net #4 have any holes?

- ◯ Yes
- ◯ No

Net #4 Condition

	0	1	2	3	4	≥ 5
Holes 0.5 to 2 cm (thumb size)	◯	◯	◯	◯	◯	◯
Holes 2 to 10 cm (bigger than thumb, smaller than fist)	◯	◯	◯	◯	◯	◯
Holes 10 to 25 cm (bigger than first, smaller than head)	◯	◯	◯	◯	◯	◯
Holes >25 cm (head size)	◯	◯	◯	◯	◯	◯

For Net #5, what was the source of this net?

- ◯ Received from government distribution
- ◯ Received at a health facility
- ◯ Purchased from store
- ◯ Other

If Net #5 was obtained from another source, please specify:

____

For Net #5, how old is this net:

ASK PARTICIPANT AND ATTEMPT TO CONFIRM ON TAG

- ◯ Less than one year old
- ◯ One to two years old
- ◯ More than two years old

Does Net #5 have any holes?

- ◯ Yes
- ◯ No

Net #5 Condition

	0	1	2	3	4	≥ 5
Holes 0.5 to 2 cm (thumb size)	◯	◯	◯	◯	◯	◯
Holes 2 to 10 cm (bigger than thumb, smaller than fist)	◯	◯	◯	◯	◯	◯
Holes 10 to 25 cm (bigger than first, smaller than head)	◯	◯	◯	◯	◯	◯
Holes >25 cm (head size)	◯	◯	◯	◯	◯	◯

For Net #6, what was the source of this net?

- ◯ Received from government distribution
- ◯ Received at a health facility
- ◯ Purchased from store
- ◯ Other

If Net #6 was obtained from another source, please specify:

____

For Net #6, how old is this net:

ASK PARTICIPANT AND ATTEMPT TO CONFIRM ON TAG

- ◯ Less than one year old
- ◯ One to two years old
- ◯ More than two years old

Does Net #6 have any holes?

- ◯ Yes
- ◯ No

Net #6 Condition

	0	1	2	3	4	≥5
Holes 0.5 to 2 cm (thumb size)	◯	◯	◯	◯	◯	◯
Holes 2 to 10 cm (bigger than thumb, smaller than fist)	◯	◯	◯	◯	◯	◯
Holes 10 to 25 cm (bigger than fist, smaller than head)	◯	◯	◯	◯	◯	◯
Holes >25 cm (head size)	◯	◯	◯	◯	◯	◯

Malaria Care Seeking

In this section, we are interested in understanding how the primary caregiver of children would seek care for fever and/or malaria. For the purposes of this section, please consider a child to be less than 18 years of age.

If a child in the household had a fever, where would the caregiver take the child for care?

- ◯ Government health center
- ◯ Private health center
- ◯ Drug shop
- ◯ VHT
- ◯ Traditional healer (i.e. herbs)
- ◯ Other

If the participant would seek care elsewhere, please specify:

____

If a government health center, which one?	◯ Mukathi Health Centre III<br>◯ Maliba Integrated Health Centre III<br>◯ Isule Health Centre III<br>◯ Kihyo Health Centre II<br>◯ Kisojo Health Centre II<br>◯ Bikoni Health Centre II<br>◯ Kenya Health Centre II<br>◯ Other
If another government health center, please specify:	________________________________
In the last three months, have you taken your child to any of the following for fever?	☐ Government health center<br>☐ Private health center<br>☐ Drug shop<br>☐ VHT<br>☐ Traditional healer (i.e. herbs)<br>☐ Other<br>(Check all that apply)
If care was pursued elsewhere, please specify:	________________________________
In the last year has any child in this household been admitted (stayed overnight) to a health clinic or hospital for malaria?	◯ Yes<br>◯ No
In the last year, has any child in this household received intravenous medication (i.e. given through a vein) for malaria	◯ Yes<br>◯ No
If the child has received IV medication for malaria, where did they receive the medicine?	◯ Hospital<br>◯ Clinic<br>◯ Drug Shop<br>◯ Other
If the child received IV medication elsewhere, please specify:	________________________________
Has any child from this household ever died from malaria?	◯ Yes<br>◯ No
If a child has died from malaria, how old was the child at the time of death?	__________________________
