# Supplementary material for: Targeted distribution of long-lasting insecticidal nets by community health workers to sustain household coverage: A pilot feasibility study in Western Uganda
Source: PLOS Glob Public Health. 2025 Jan 24;5(1):e0003660. doi: 10.1371/journal.pgph.0003660 (PMC11759381; doi:10.1371/journal.pgph.0003660)
Supplement: S2 Appendix — (PDF) [file pgph.0003660.s006.pdf]

Post-Intervention

REDCap #	______________________ (Automatic Pull)
Village	◯ Kateebe 1<br>◯ Nyarukungu
Study Number	______________________
Study Number	______________________
Does the study number match?	◯ Yes<br>◯ No
Date of Visit	______________________
RA Documenting Consent	◯ Mugisa Saul<br>◯ Kibaba Georget<br>◯ Bwambale Jonathan<br>◯ Bwambale Aprunale<br>◯ Biira Doreen<br>◯ Asikibawe Norbert<br>◯ Lyhinda Francis<br>◯ Baluku Jockim
Comments	____________________________

Post-Intervention_LLIN Survey

REDCap # ______________________ (Automatic Pull)

Insecticide Treated Nets

Does your household have any mosquito nets?	◯ Yes<br>◯ No
How many mosquito nets does your household have?	______________________
How many mosquito nets were used last night?	______________________
How many household members slept under a mosquito net last night?	______________________
Did any household members NOT sleep under a mosquito net last night?	◯ Yes<br>◯ No
Please list the age and sex of any household members who did NOT sleep under a mosquito net last night	______________________
Why did these members not sleep under a net?	☐ Not enough nets available<br>☐ Net has too many holes or is damaged<br>☐ Too hot, uncomfortable, or don't like smell<br>☐ No place to hang or place it<br>☐ Not many mosquitoes / Low risk for malaria<br>☐ Other<br>(Select all that apply)
If other reasons stated for not using a mosquito net, please list here:	______________________
For Net #1, what was the source of this net?	◯ Received from government distribution<br>◯ Received at a health facility<br>◯ Purchased from store<br>◯ Other
If Net #1 was obtained from another source, please specify:	______________________
For Net #1, how old is this net:<br><br>ASK PARTICIPANT AND ATTEMPT TO CONFIRM ON TAG	◯ Less than one year old<br>◯ One to two years old<br>◯ More than two years old
Does Net #1 have any holes?	◯ Yes<br>◯ No

Net #1 Condition

	0	1	2	3	4	≥ 5
Holes 0.5 to 2 cm (thumb size)	◯	◯	◯	◯	◯	◯
Holes 2 to 10 cm (bigger than thumb, smaller than fist)	◯	◯	◯	◯	◯	◯
Holes 10 to 25 cm (bigger than first, smaller than head)	◯	◯	◯	◯	◯	◯
Holes >25 cm (head size)	◯	◯	◯	◯	◯	◯

For Net #2, what was the source of this net?

◯ Received from government distribution
◯ Received at a health facility
◯ Purchased from store
◯ Other

If Net #2 was obtained from another source, please specify:

____

For Net #2, how old is this net:

ASK PARTICIPANT AND ATTEMPT TO CONFIRM ON TAG

◯ Less than one year old
◯ One to two years old
◯ More than two years old

Does Net #2 have any holes?

◯ Yes
◯ No

Net #2 Condition

	0	1	2	3	4	≥ 5
Holes 0.5 to 2 cm (thumb size)	◯	◯	◯	◯	◯	◯
Holes 2 to 10 cm (bigger than thumb, smaller than fist)	◯	◯	◯	◯	◯	◯
Holes 10 to 25 cm (bigger than first, smaller than head)	◯	◯	◯	◯	◯	◯
Holes >25 cm (head size)	◯	◯	◯	◯	◯	◯

For Net #3, what was the source of this net?

◯ Received from government distribution
◯ Received at a health facility
◯ Purchased from store
◯ Other

If Net #3 was obtained from another source, please specify:

____

For Net #3, how old is this net:

ASK PARTICIPANT AND ATTEMPT TO CONFIRM ON TAG

◯ Less than one year old
◯ One to two years old
◯ More than two years old

Does Net #3 have any holes?

◯ Yes
◯ No

Net #3 Condition

	0	1	2	3	4	≥ 5
Holes 0.5 to 2 cm (thumb size)	◯	◯	◯	◯	◯	◯
Holes 2 to 10 cm (bigger than thumb, smaller than fist)	◯	◯	◯	◯	◯	◯
Holes 10 to 25 cm (bigger than first, smaller than head)	◯	◯	◯	◯	◯	◯
Holes >25 cm (head size)	◯	◯	◯	◯	◯	◯

For Net #4, what was the source of this net?

◯ Received from government distribution
◯ Received at a health facility
◯ Purchased from store
◯ Other

If Net #4 was obtained from another source, please specify:

____

For Net #4, how old is this net:

ASK PARTICIPANT AND ATTEMPT TO CONFIRM ON TAG

◯ Less than one year old
◯ One to two years old
◯ More than two years old

Does Net #4 have any holes?

◯ Yes
◯ No

Net #4 Condition

	0	1	2	3	4	≥ 5
Holes 0.5 to 2 cm (thumb size)	◯	◯	◯	◯	◯	◯
Holes 2 to 10 cm (bigger than thumb, smaller than fist)	◯	◯	◯	◯	◯	◯
Holes 10 to 25 cm (bigger than first, smaller than head)	◯	◯	◯	◯	◯	◯
Holes >25 cm (head size)	◯	◯	◯	◯	◯	◯

For Net #5, what was the source of this net?

◯ Received from government distribution
◯ Received at a health facility
◯ Purchased from store
◯ Other

If Net #5 was obtained from another source, please specify:

____

For Net #5, how old is this net:

ASK PARTICIPANT AND ATTEMPT TO CONFIRM ON TAG

◯ Less than one year old
◯ One to two years old
◯ More than two years old

Does Net #5 have any holes?

◯ Yes
◯ No

Net #5 Condition

	0	1	2	3	4	≥ 5
Holes 0.5 to 2 cm (thumb size)	◯	◯	◯	◯	◯	◯
Holes 2 to 10 cm (bigger than thumb, smaller than fist)	◯	◯	◯	◯	◯	◯
Holes 10 to 25 cm (bigger than first, smaller than head)	◯	◯	◯	◯	◯	◯
Holes >25 cm (head size)	◯	◯	◯	◯	◯	◯

For Net #6, what was the source of this net?

◯ Received from government distribution
◯ Received at a health facility
◯ Purchased from store
◯ Other

If Net #6 was obtained from another source, please specify:

____

For Net #6, how old is this net:

ASK PARTICIPANT AND ATTEMPT TO CONFIRM ON TAG

◯ Less than one year old
◯ One to two years old
◯ More than two years old

Does Net #6 have any holes?

◯ Yes
◯ No

Net #6 Condition

	0	1	2	3	4	≥5
Holes 0.5 to 2 cm (thumb size)	◯	◯	◯	◯	◯	◯
Holes 2 to 10 cm (bigger than thumb, smaller than fist)	◯	◯	◯	◯	◯	◯
Holes 10 to 25 cm (bigger than fist, smaller than head)	◯	◯	◯	◯	◯	◯
Holes >25 cm (head size)	◯	◯	◯	◯	◯	◯

Post-Intervention Survey

REDCap #	______________________ (Automatic Pull)
Did you participate in the first survey?	◯ Yes<br>◯ No
Has anyone in your household tested positive (by RDT or smear) for malaria since the last survey?	◯ Yes<br>◯ No
If yes, was it an adult or child?	◯ Adult<br>◯ Child (< 18 years old)
Did you get any new bed nets since the last time we visited?<br><br>Any source including purchases from a shop, distributed from clinics or by VHTs	◯ Yes<br>◯ No
Did you receive any new bed nets from a VHT since the last visit?	◯ Yes<br>◯ No
If so, how many nets did you receive?	______________________
Has anyone in your household had malaria since you received additional nets from the VHT?	◯ Yes<br>◯ No
How satisfied are you with the number aof nets you have available in your house?	◯ Completely satisfied - We do not need any more nets ◯ Satisfied - We would like more nets, but it's not a high priority OR we probably wouldn't use them regularly<br>◯ Partly satisfied - We need more nets, but it doesn't have a major impact on our life<br>◯ Not satisfied - We need more nets and the lack of nets means we get more malaria
How satisfied are you with the condition aof nets you have available in your house?	◯ Completely satisfied - Our nets are in good shape ◯ Satisfied - Our nets have a few holes or tears, but it does not have a major impact ◯ Partly satisfied - Our nets have large holes that sometimes let mosquitoes in<br>◯ Not satisfied - Our nets are so damaged that we often don't use them or stopped using them ◯ N/A - We don't have any nets

Post-Intervention_Testing

REDCap #

____
(Automatic Pull)

Household Members

Include any individuals who spend most nights of the week in the house.

How many individuals live in this household?

____

How many adults (age ≥ 18 years) live in this household?

____

How many older children (age 13 to 18 years) live in this household?

____

How many children (age 5 to 12 years) live in this household?

____

How many children (age less than 5 years) live in this household?

____

Child Information

Include information on all children 17 years and younger who live in the household. Enter data from youngest child to oldest child. For example, Child #1 should be the oldest child.

If more than 6 children live in the household, document additional information in the Comments section below.

Sex of Child #1 (Oldest)

- ◯ Female
- ◯ Male

Age of Child #1

IF CHILD IS LESS THAN 1 YEAR, ENTER 0 WHICH WILL OPEN NEW QUESTION ABOUT MONTHS

____
(Years)

If Child #1 is less than 1 year of age, how many months old is the child

____
(Months)

Did Child #1 sleep under a bed net last night?

- ◯ Yes
- ◯ No

Child #1 temperature:

____
(Degrees Celsius)

Child #1 Mid-Upper Arm Circumference (MUAC)<br><br>ONLY FOR CHILDREN 2 to 5 YEARS OF AGE	____________________<br>(centimeters)
Child #1 Malaria RDT Result:	◯ Negative<br>◯ Positive<br>◯ Invalid / Unable to Perform
Sex of Child #2	◯ Female<br>◯ Male
Age of Child #2<br><br>IF CHILD IS LESS THAN 1 YEAR, ENTER 0 WHICH WILL OPEN NEW QUESTION ABOUT MONTHS	____________________<br>(Years)
If Child #2 is less than 1 year of age, how many months old is the child	____________________<br>(Months)
Did Child #2 sleep under a bed net last night?	◯ Yes<br>◯ No
Child #2 temperature:	____________________<br>(Degrees Celsius)
Child #2 Mid-Upper Arm Circumference (MUAC)<br><br>ONLY FOR CHILDREN 2 to 5 YEARS OF AGE	____________________<br>(centimeters)
Child #2 Malaria RDT Result:	◯ Negative<br>◯ Positive<br>◯ Invalid / Unable to Perform
Sex of Child #3	◯ Female<br>◯ Male
Age of Child #3<br><br>IF CHILD IS LESS THAN 1 YEAR, ENTER 0 WHICH WILL OPEN NEW QUESTION ABOUT MONTHS	____________________<br>(Years)
If Child #3 is less than 1 year of age, how many months old is the child	____________________<br>(Months)
Did Child #3 sleep under a bed net last night?	◯ Yes<br>◯ No
Child #3 temperature:	____________________<br>(Degrees Celsius)
Child #3 Mid-Upper Arm Circumference (MUAC)<br><br>ONLY FOR CHILDREN 2 to 5 YEARS OF AGE	____________________<br>(centimeters)

Child #3 Malaria RDT Result:	◯ Negative<br>◯ Positive<br>◯ Invalid / Unable to Perform
Sex of Child #4	◯ Female<br>◯ Male
Age of Child #4<br><br>IF CHILD IS LESS THAN 1 YEAR, ENTER 0 WHICH WILL OPEN NEW QUESTION ABOUT MONTHS	______________<br>(Years)
If Child #4 is less than 1 year of age, how many months old is the child	______________<br>(Months)
Did Child #4 sleep under a bed net last night?	◯ Yes<br>◯ No
Child #4 temperature:	______________<br>(Degrees Celsius)
Child #4 Mid-Upper Arm Circumference (MUAC)<br><br>ONLY FOR CHILDREN 2 to 5 YEARS OF AGE	______________<br>(centimeters)
Child #4 Malaria RDT Result:	◯ Negative<br>◯ Positive<br>◯ Invalid / Unable to Perform
Sex of Child #5	◯ Female<br>◯ Male
Age of Child #5<br><br>IF CHILD IS LESS THAN 1 YEAR, ENTER 0 WHICH WILL OPEN NEW QUESTION ABOUT MONTHS	______________<br>(Years)
If Child #5 is less than 1 year of age, how many months old is the child	______________<br>(Months)
Did Child #5 sleep under a bed net last night?	◯ Yes<br>◯ No
Child #5 temperature:	______________<br>(Degrees Celsius)
Child #5 Mid-Upper Arm Circumference (MUAC)<br><br>ONLY FOR CHILDREN 2 to 5 YEARS OF AGE	______________<br>(centimeters)
Child #5 Malaria RDT Result:	◯ Negative<br>◯ Positive<br>◯ Invalid / Unable to Perform

Sex of Child #6	◯ Female<br>◯ Male
Age of Child #6<br><br>IF CHILD IS LESS THAN 1 YEAR, ENTER 0 WHICH WILL OPEN NEW QUESTION ABOUT MONTHS	________________<br>(Years)
If Child #6 is less than 1 year of age, how many months old is the child	________________<br>(Months)
Did Child #6 sleep under a bed net last night?	◯ Yes<br>◯ No
Child #6 temperature:	________________<br>(Degrees Celsius)
Child #6 Mid-Upper Arm Circumference (MUAC)<br><br>ONLY FOR CHILDREN 2 to 5 YEARS OF AGE	________________<br>(centimeters)
Child #6 Malaria RDT Result:	◯ Negative<br>◯ Positive<br>◯ Invalid / Unable to Perform
Additional Comments	____________________
