# Supplementary material for: Targeted distribution of long-lasting insecticidal nets by community health workers to sustain household coverage: A pilot feasibility study in Western Uganda
Source: PLOS Glob Public Health. 2025 Jan 24;5(1):e0003660. doi: 10.1371/journal.pgph.0003660 (PMC11759381; doi:10.1371/journal.pgph.0003660)
Supplement: Attachment — Submitted filename: Bednet Response to Reviewers.docx [file pgph.0003660.s008.docx]

July 23, 2024

Abhinav Sinha, M.D.

Academic Editor
*PLOS Global Public Health*

RE: [PGPH-D-24-01366] - [EMID:22b0edaa6d66f100] – Response to reviewer comments

Dear Dr. Sinha:

Thank you for the review of our manuscript entitled, “**Targeted distribution of long-lasting insecticidal nets by community health workers to sustain household coverage: a pilot feasibility trial in western Uganda.**” We appreciate the detailed review and believe that the revised manuscript is much improved. Please find our responses (in blue) to reviewers’ comments below:

**REVIEWER 1**

**Overall**: I have reviewed the manuscript titled "Targeted distribution of long-lasting insecticidal nets by community health workers to sustain household coverage: a pilot feasibility trial in western Uganda" and appreciate the thorough investigation into the implementation and impact of community health worker-led interventions. The authors' exploration of the feasibility and sustainability of targeted long-lasting insecticidal net distribution offers significant insights for public health practitioners and policymakers aiming to enhance malaria prevention strategies. However, several important issues require attention.

**Response**: We greatly appreciate the time and effort given to review this manuscript and have addressed each comment below.

**Title**

**Comment 0.1**: The study does not exhibit the characteristics of a trial. It is recommended to use "pilot feasibility study" instead of "pilot feasibility trial" in the title.

**Response**: We agree with the suggested edit and have amended the title.

**Introduction**

**Comment 1.1**: The malaria data presented in the introduction is outdated. Please update the data with the most recent malaria reports.

**Response**: We reviewed the World Malaria Report from 2023 and amended the citation. The percentages were consistent between reports.

**Comment 1.2**: In the last line of the introduction paragraph, it is unclear whether the statement refers to Uganda specifically or to the global population in general. Please clarify and verify reference number 4.

**Response**: The text was amended to specify that Uganda is specifically being referred to (line 75). Reference 4 in the original draft was modified to specifically reference the 2024 plan for Uganda and was moved to reference 7 in the revised manuscript on line 82 where it provides more direct support. A new citation was added to the last line of the first paragraph (line 75) which provides more direct support for the current trends and state of malaria in Uganda.

**Comment 1.3**: In paragraph 2, line 77, a more recent citation should be provided to highlight the effectiveness of long-lasting insecticidal nets (LLIN) against malaria.

**Response**: We updated this citation with an article published in 2022 using durability and efficacy data from the most recent mass distribution campaign (citation 4, line 77).

**Comment 1.4**: A reference for the WHO universal coverage statement is missing. According to WHO, "Universal coverage for malaria vector control is defined as universal access to and use of appropriate interventions by populations at risk of malaria." The term "universal coverage" is not suitable here and should be replaced with a more appropriate term. The current coverage rate, including accessibility and utilization attrition rate, and loss rate in Uganda should also be presented.

**Response**: Thank you for this comment. We reviewed the “WHO recommendations for achieving universal coverage with long-lasting insecticidal nets in malaria control” published in 2017. This document includes the ratio of one LLIN per two people living in a household as part of the recommendations for mass distribution campaigns. The term “universal coverage” is also used throughout the literature to refer to ratio as a threshold for reaching universal access, therefore, we believe the term is appropriately used. We have added a citation for this report to the paper.

**Study Aim and Methodology**

**Comment 2.1**: The aim of the study provided does not align with the methodology and results sections. The LLIN coverage gap in Uganda should be explained in detail, referencing published literature. Additionally, the reduction of malaria transmission is not mentioned in the results or discussion sections and should be revised accordingly.

**Response**: We amended the description of the study aims to (lines 96 to 104):

“The main aim of this work was to address the LLIN coverage gap that emerges in the period after mass distribution campaigns and thereby reduce malaria transmission through implementation of a novel LLIN distribution strategy leveraging CHWs carrying out iCCM. As a first step towards this overall goal, this pilot study focused on assessing the feasibility and additional burden of integrating LLIN distribution into the current tasks of CHWs as well as understanding the level of need in the community.”

**Comment 2.2**: In the methodology, it is unclear how both the intervention and control villages were selected. The criteria for their selection need to be clearly explained.

**Response**: The two villages enrolled were selected based on participation in the iCCM program supported by our research team, giving access to monthly data including the number of children assessed for malaria and diagnosed with malaria. Further, previous research conducted in the adjacent Bugoye sub-county indicated a PfPR among children 2 to 10 years of age over 30% for villages with similar terrain and geography as those selected for this study. Based on previous iCCM data, Kateebe 1 and Nyarukungu, had similar population sizes, number of iCCM visits, and proportion iCCM visits attributable to malaria during the previous year. This text was added to the manuscript on line 137.

**Comment 2.3**: The status of Nyagorongo and Bhugunga, from where the intervention and control villages were selected, should be explained. The reasons for choosing these areas, considering their socio-economic, ecological, and ethnic differences, as shown in Table 1, should be provided.

**Response**: The main goal of this study was to assess the feasibility of implementing the intervention in a larger study and not assess causality as in a full RCT. Therefore, these villages were not selected to minimize differences between them but were selected more by convenience and results should not be overinterpreted.

However, we did add additional information and commentary about the differences between the two villages to the discussion section (line 434):

“Secondly, these two communities were selected as a convenience sample and not balanced by potential covariates of interest. Therefore, noticeable differences were seen with the intervention village having a higher overall SES and a lower incidence rate of malaria and malaria related hospitalizations than the control village. Further, the control village was located at a lower elevation and in closer proximity to a stream. While we do not believe these factors had an impact on assessing feasibility, it is possible that more LLINs would have been distributed in the control village had it been designated the intervention village, conveying a greater need for the novel intervention but possibly overstating the impacts of the program on reductions in malaria cases."

**Comment 2.4**: It is also unclear why a control village was included when the objective was to assess the feasibility of interventions by community health workers (CHWs). This should be justified.

**Response**: This study was intended to be the first step to a larger trial that will include multiple control and intervention villages. Therefore, we wanted to pilot the methods that would be used in the future study to understand how the community members would respond to CHWs in control setting where they were not handing out nets.

**Comment 2.5**: The gap identified in the Malaria Indicator Survey (MIS) should be part of the introduction to justify the research question. Additionally, the PfPr rates for Maliba sub-county should be provided to support the study site selection.

**Response**: We have not been able to identify data as granular as the sub-county level or any available estimates for Maliba sub-county available from any official sources as this study was conducted in a rural, under-served area of Uganda. Given this gap, we have provided contextual data for the district level as seen on line 133. We further provided additional context for Bugeye sub-county based on our group’s previous research, which directly boarders Maliba sub-county. We added some additional clarification for the relationship between the two sub-counties mentioned in line 134.

**Intervention Section**

**Comment 3.1**: The criteria for site selection should be clearly written.

**Response**: Please see our response to comment 2.2

**Comment 3.2**: Figure 2 lacks a clear timeline, including community sensitization, pre-intervention survey, CHW training, iCCM performance, LLIN assessment and distribution, post-intervention survey, and interviews with CHWs (FGDs). These details, including the timeline (dates), human resources involved, and monitoring details, are crucial for understanding the feasibility of the interventions.

**Response**:

Dates were added to Figure 2 to make clear the timeline of study events.

To provide clarity for resources, we added the following text to line 196:

“For this study eight members of the nearby health facilities were trained over the course of two days to serve as research assistants and carry out data collection (150,000 Ugandan Shillings per day). They assisted in a day long training CHWs covering how to calculate the number of LLINs to distribute to each household and how to communicate with families about eligibility to receive LLINs. Minimal resources were needed to carry out this intervention, in addition to procurement of the LLINs, including stationery for data entry forms and calculations, meals and transportation for the training, and financial support for cell phone payments. While CHWs were provided a monthly allowance (20,000 Ugandan Shillings each) for their participation in the study, CHWs in the area work as volunteers.”

**Comment 3.3**: The questionnaire used for the pre-intervention and post-intervention surveys should be provided.

**Response**: Both the pre- and post-intervention surveys have been added as appendix S1 and appendix S2, respectively. They are referenced on line 188.

**Comment 3.4**: Despite the feasibility study design, the sample size for recruiting households (HHs) in both intervention and control arms should be calculated.

**Response**: Due to the pilot nature of the study, no power calculations were conducted for the primary or secondary

outcomes. We feel that conducting a power calculation at this time would not provide additional benefit to the study. Results from this study will be used to inform power calculations for the larger planned study.

**Comment 3.5**: In qualitative data collection, the number of CHWs involved in FGDs, the number of FGDs conducted in intervention and control villages, and whether they were IDIs or FGDs should be clearly detailed.

**Response**: We conducted an in-depth interview with each of the four participating (two from each village) to get feedback and gauge their perceptions of the program. These questions informed the progress check and feedback during the project focusing on the following topics:

1. Workload to distribute LLINs as part of iCCM
2. Impressions of community need and acceptance of the program
3. Perceptions about barriers to implementation at scale

We made the following edits to the Qualitative Data Collection section on line 207:

“After study follow-up concluded, an in-depth interview was conducted with each of the four participating CHWs, two from each village to better understand the community’s acceptance to LLIN distribution and ease of calculations for LLIN distribution. In the intervention village additional questions about the burden and challenges associated with the intervention were asked as well as potential challenges with scaling up the intervention.”

**Outcome Indicators**

**Comment 4.1**: For the primary outcome indicator (proportion of iCCM clients (HH) eligible to receive LLIN), specify if there were any cutoffs. Similarly, for secondary outcome indicators, provide the cutoffs or criteria to check feasibility and acceptability.

**Response**: The only variable using any cutoffs was the universal coverage variable. All other variables were continuous measures of count or proportion data. The following text was added to the analysis section to provide additional clarity (line 224):

“All variables were measured as continuous variables other than status of households regrading universal coverage which was measured as a three-level categorical variable defined as owning no LLINs, owning LLINs but not enough to reach the universal coverage threshold, and owning enough LLINs to reach the universal coverage threshold.”

In relation to checking feasibility and acceptability, the main metrics came from interviews with CHWs regarding their experience with implementation. No specific cutoff was outlined for the number of LLINs distributed but we feel that the data are clear on the level of need in the communities. In the larger study we have planned, we intend to set clearer cutoffs.

**Results Section**

**Comment 5.1**: It is unclear why the authors considered the age of children as 5-18 years in the results section while testing for iCCM included 2-18 years. The pre-intervention survey considered data for below 18 years, whereas the outcome indicator was for families with 2-10-year-old malaria-positive cases. Clarify if there were significant differences in malaria cases between case and control villages and why they were considered for inclusion.

**Response**: CHWs as part of iCCM, at least in our study area, only see children under 5 years old and not 2 to 18 year old. Our data collection of anthropometric measures includes children up to the age of 12 as we believe that children older than 5 can still play an important role in transmission. It is unclear what section of the results are being referred to in this comment as pertaining to children aged 5 to 18. There are inconstancies in the age ranges of children being tested for malaria by the study (2 to 10) and those who had their MUAC measured (2 to 5). These differences are mainly due to oversight in the study design and have since been changed in our data collection procedures.

We did not perform significance testing regarding malaria cases between the intervention and control villages due to a small sample size and lack of statistical power. We feel that running tests of significance would be an over-interpretation of the findings.

**Comment 5.2**: It is unclear how 100 LLINs covered 53 malaria-positive children HHs with missing and short LLINs per HH. Similarly, explain how all eligible HHs achieved universal coverage when 9 eligible children HHs could not receive LLINs.

**Response**: We distributed more than one LLIN to a house if they required more than one LLIN to reach universal coverage, therefore, the 100 LLINs did not cover 100 households. We added the following text to line 284:

“All 100 LLINs were distributed to 53 households of the 62 eligible to receive LLINs while the remaining 9 children eligible did not receive LLINs due to shortages. Multiple LLINs were given to each household as needed to reach universal coverage. Therefore, the 100 LLINs from the study did not cover 100 households.”

The following statement was misleading and removed from the manuscript:

“All eligible households in the intervention village were brought to universal coverage.”

**Comment 5.3**: In pre and post-intervention statistics, explain why fewer HHs were followed for the post-intervention survey.

**Response**: We added the following sentence to line 310 for additional clarity.

“Not all households surveyed in the pre-intervention wave were reached in the post-intervention survey most often because household members were not present at the home when visited by research teams during data collection or additional follow-up attempts.”

We also added the following sentence to line 169 to clarify the data collection methods. Study staff did not follow households longitudinally, instead we conducted two separate surveys with their own consent processes and longitudinal records kept by CHWs for the households that gave consent during the first survey. The discrepancy is because less households were able to be reached in the second wave of the survey.

“Data were collected using two cross-sectional surveys before and after a three-month period of data collection by CHWs with the potential for households to appear in either the pre-intervention survey, post-intervention survey, or both surveys.”

**Comment 5.4**: When almost all HHs were covered with LLIN distribution, clarify why only 6.5% of HHs met universal coverage.

**Response**: We are making the assumption that the reviewer is referring to table 3. Here there are only 12 households at universal coverage in the intervention village at the post-intervention survey. This number is lower than expected given households for 53 children were given LLINs to reach universal coverage. We hypothesize that over the study period, any LLINs that were owned by the families may have become un-usable after they were given new LLINs or that families opted not to use their older LLINs in favor of just using the newer LLINs. We do expect that over the three months that there was movement between the categories as the LLINs from the national distribution would have been about 4 years old and fairly deteriorated.

The following text was added to the manuscript on line 377:

“It is notable that 12 households were found to reach the universal coverage threshold during the post-intervention survey in the intervention village although 29 households reported receiving LLINs from the study. We hypothesize that over the study period, there was continued attrition of LLINs from the previous national distribution four years earlier and that the loss of those LLINs could have led to households falling back below the threshold. Secondly, it is possible that many of the households receiving LLINs from the study were only captured in the pre-intervention survey, estimated to be 24 households, leading to over representation of those below the threshold.”

**Comment 5.5**: Table 3 is unclear, particularly why after distributing LLINs in 62 malaria-positive children's households (Table 2), there were no LLINs in 11 households in the post-intervention survey, and only 12 HHs had universal coverage.

**Response**: We have verified that there are no discrepancies between the table and the data. Please see comment 5.4

**Comment 5.6**: In Table 3, the data in columns 13 and 14 is confusing. When the PfPr among children 2-10 years of age is 9%, clarify how the household reporting incident malaria infection at the pre-intervention is zero. Revise the data presentation of malaria rates in households pre and post-interventions for better clarity.

**Response**: We are assuming that the comment is referring to the “Households reporting incident malaria infection during study period (any households)” row and “PfPR among children 2-10 years of age” row. Regarding the comment about the PfPR of 9%, the PfPR for the pre-intervention survey is the prevalent cases at the time of the survey. The row above says “NA”, not 0 because as it is referring to cases over the course of the study period. Therefore, there are no incident cases to be reported at the pre-intervention time point since no follow-up has occurred (NA – not applicable). We then have 58 (54.2) listed for the post-intervention survey to denote that it is measured over the course of the study. We did amend the row label in the table to say “Prevalent PfPR among children 2-10 years of age” to better contrast the term incident used in the other label.

**Comment 5.7**: Present the qualitative data thematically.

**Response**: We added two sub-headings “Burden of work” and “Challenges.”

**Discussion**

**Comment 6.1**: Revisit and discuss the feasibility and acceptability claims with primary outcome indicator data.

**Response**: The following text was added to line 360:

“Of the children who tested positive for malaria, 100% and 96% of the children in the intervention and control villages were eligible to receive at least on LLIN, showing the need to additional LLIN distribution. Further, the complete distribution of LLINs given to the CHWs for this study indicates the feasibility of this method but may require additional effort to ensure the LLIN with CHWs remains sufficient.”

**Comment 6.2**: Explain what "algorithm-based care" entails.

**Response**: We added the additional phrase to line 386 for greater clarity.

“Not only are these individuals experienced with providing algorithm-based care of young children under the iCCM program**, through the use of clear flowcharts for diagnosis and treatment,** but they are also trusted members of local communities (20).”

**Comment 6.3**: The acceptability of interventions in the community is not properly assessed. This should be discussed in light of the results.

**Response**: We added the following text to the limitations section on line 428:

“Due to the limitations of the pilot design, we did not have the resources to carry out larger focus groups with community members and instead relied on reports made to the CHWs. Therefore, we were not able to fully assess community sentiments about the program directly.”

**REVIEWER 2**

**Comment 1**: The net result is that in the intervention village there was a two-time increase in the usage of nets, whereas in the control village the same was reversed with huge decrease in the usage. Please explain the reasons.

**Response**: Thank you for this comment. We added the following text to line 371 in the discussion:

“We saw a one-fold increase in LLIN usage in children under 5 in the intervention village and a two-fold decrease in the control village, with similar directional trends in medians for the overall number of household members sleeping under an LLIN. This increase is likely driven by increased access and quality of LLINs in the home through the intervention while LLINs continued to deteriorate in the control village.”

**Comment 2.1**: There is no information on the attrition rate.

**Response**: The households in each survey were not followed by our study team through time, rather data was collected during two cross-sectional surveys, and we obtained data from the CHWs over the three-month period between surveys. Therefore, we do not feel like there is a true attrition rate for participation. We added the following text to the results section on line 308 to address the disparity in households between surveys:

“There was a reduction of 44 and 31 households enrolled from the post-intervention survey compared to the pre-intervention survey in the intervention and control villages, respectively.”

We further added the text seen in comment 5.3 and the following sentence to line 190 to clarify:

“This tracking included only households enrolled in at least the pre-intervention survey.”

Thank you for your continued consideration of our manuscript.

Kind regards,

Ross M. Boyce MD, MSc

Assistant Professor

Division of Infectious Diseases
