# Supplementary material for: Targeted distribution of long-lasting insecticidal nets by community health workers to sustain household coverage: A pilot feasibility study in Western Uganda
Source: PLOS Glob Public Health. 2025 Jan 24;5(1):e0003660. doi: 10.1371/journal.pgph.0003660 (PMC11759381; doi:10.1371/journal.pgph.0003660)
Supplement: Attachment — Submitted filename: LLIN_R2R2.docx [file pgph.0003660.s009.docx]

December 10, 2024

Abhinav Sinha, M.D.

Academic Editor
*PLOS Global Public Health*

RE: [PGPH-D-24-01366] - [EMID:22b0edaa6d66f100] – Response to reviewer comments

Dear Dr. Sinha:

Thank you for the review of our manuscript entitled, “**Targeted distribution of long-lasting insecticidal nets by community health workers to sustain household coverage: a pilot feasibility trial in western Uganda.**” We appreciate the detailed review and believe that the revised manuscript is much improved. Please find our responses (in blue) to reviewers’ comments below:

**REVIEWER 1**

**Overall**: I appreciate the revisions made to the manuscript, and I found that most of my previous comments have been addressed satisfactorily. However, I would like to raise a few additional points for clarification:

**Response**: We greatly appreciate the time and effort given to a second round of reviews this manuscript and have addressed each comment below.

**Comment 1**: I observed significant variability in the sample sizes presented across Tables 1, 2, and 3. This inconsistency makes it challenging to understand the reasons behind the differences. For instance, in Table 1, the total number of children is 488 in 154 households, whereas in Table 3, the number of children is 474 in 153 households. It is unclear why these figures do not match. Could you kindly clarify whether these households are the same or if they differ between Table 1 and Table 3? Additionally, the sample sizes for pre-intervention households in both intervention and control villages (table 3) do not align with the data provided in Table 1. A detailed explanation for these discrepancies would be helpful. Furthermore, please ensure that the table names are placed above the tables for consistency.

**Response**: We want to highlight that the pre- and post-intervention surveys were cross-sectional in design. In other words, we did not follow the same households longitudinally. This introduced some variance between the surveys as not all of the same households were surveyed (e.g., an adult household member not present during post-intervention survey). We have made the following revisions to present the results more clearly:

The table titles were moved to be above the tables. Discrepancies for the sample size in Table 2 are addressed in the comment below. For Table 3, the following footnote was added to add clarity:

“^1^ 14 children from the intervention village and 4 children from the control village were excluded due to missing data for malaria related variables”

Regarding Table 1, the analysis was redone, and the household totals are 153 and 180. These totals have been corrected. Percentages were calculated using the correct totals and were, therefore, unchanged. Supplemental Table 1 was also updated.

**Comment 2**: I noticed that there is a discrepancy between the numbers mentioned in the text and those presented in Table 2. This should be addressed in the manuscript. It would be beneficial if the text explicitly references the corresponding table numbers to avoid confusion.

**Response**: We double checked all the numbers presented for table 2 and they do match the numbers in the text. The text included some percentages alongside the counts which were omitted from the table to reduce confusion about the denominator. We did add a reference to table 2 in two additional locations in lines 286 and 302.

We added the following text to the caption for table 2 to reduce confusion over the inconsistency of total children we counted in the villages compared to those seen by the CHWs with the underlined portion of the text representing the addition:

“^1^ The number of children enumerated by the study may be an undercount of children living in the communities. CHWs recorded more children in the village than were included in our baseline survey, which is why these numbers do not match the total number of children seen by a CHW for any iCCM condition. Children from other villages and repeat visits were excluded from CHW counts.”

**Comment 3**: In Table 3, the sample size for the intervention village's post-intervention households is listed as 109, but in the LLIN coverage data, the figure provided is for 144 households. None of these totals align with the household numbers provided for either the intervention or control groups.

**Response**: We are assuming that you are referring to the number “140” noted at the top of the care cascade for the intervention village (supplemental figure 2). This number of the total number of people we enumerated for the village in table 2 as discussed in the previous comment’s response. Table 3 uses data from the pre- and post-intervention surveys while these supplemental figures use the longitudinal CHW visits. We added the following text to line 305 to clarify which table the figures correspond to:

“Care-cascades are also represented in Supplemental Figures 1 and 2 for the intervention and control villages, respectively.”

**Comment 4**: The details provided in lines 199-200 regarding funding are not necessary for the revised manuscript. I suggest omitting this information.

**Response**: The text mentioned was removed from the manuscript.

**Comment 5**: The statement “They assisted in a day-long training of CHWs covering how to calculate the number of LLINs to distribute to each household and how to communicate with families about eligibility to receive LLINs” is unclear. It is not specified who "they" refers to.

**Response**: For clarity, the term “they” was replaced with “The research assistants.”

**Comment 6**: The significant difference in sociodemographic variables, particularly the access to piped water between the control and intervention villages, could potentially contribute to the observed high malaria incidence rate. This factor can be discussed in the discussion section to provide a comprehensive analysis.

**Response**: Thank you for this comment. This connection was discussed in the discussion section starting on line 444, we did, however, add the following section of underlined text on line 447. We did not elaborate further as this study was not powered to assess malaria incidence and the overall aim was to look at the feasibility of the distribution technique and not a reduction in incidence rates.

“Secondly, these two communities were selected as a convenience sample and not balanced by potential covariates of interest. Therefore, noticeable differences were seen with the intervention village having a higher overall SES and a lower incidence rate of malaria and malaria related hospitalizations than the control village, which are likely related given the established connection between SES and malaria (34).”

**REVIEWER 2**

**Comment 1**: Still my question on LLIN attrition is not addressed properly. Because attrition rate will decide the community acceptance and ultimately the overall impact on the disease.

**Response**: As re-iterated above, given the pilot feasibility design, short intervention period, and limited funding, we elected to perform two cross-sectional surveys rather than longitudinally follow households. Many of the same households participated in both the pre-and post-survey, but we did not match them, which makes assessment of attrition difficult. We do note that this was not an objective of the study, largely because we would not expect significant changes in LLIN attrition within the limited 90-day timeframe. We added the following text to line 406 in the discussion:

“There may also be challenges maintaining the supply of LLINs if LLIN attrition rates are high. As this was a pilot feasibility study with a limited, 3-month duration, we did not assess attrition of the existing or newly provided LLINs (23–25).”

Thank you for your continued consideration of our manuscript.

Kind regards,

Ross M. Boyce MD, MSc

Assistant Professor

Division of Infectious Diseases
